# Development of a 2-(2-Hydroxyphenyl)-1*H*-benzimidazole-Based Fluorescence Sensor Targeting Boronic Acids for Versatile Application in Boron Neutron Capture Therapy

**DOI:** 10.3390/cancers15061862

**Published:** 2023-03-20

**Authors:** Naoya Kondo, Shinya Takada, Masayori Hagimori, Takashi Temma

**Affiliations:** 1 Department of Biofunctional Analysis, Graduate School of Pharmaceutical Sciences, Osaka Medical and Pharmaceutical University, 4-20-1 Nasahara, Takatsuki 569-1094, Osaka, Japan; 2 Laboratory of Analytical Chemistry, Faculty of Pharmaceutical Sciences, Mukogawa Women’s University, 11-68 Koshien Kyubancho, Nishinomiya 663-8179, Hyogo, Japan

**Keywords:** boron neutron capture therapy, boronoagents, Borofalan, fluorescence sensor

## Abstract

**Simple Summary:**

In boron neutron capture therapy (BNCT), the amount and localization of boron-10 atoms in tumor tissues directly determine the therapeutic effect. This study developed a novel fluorescence sensor, BITQ, to analyze boronic acid agents used for BNCT. Mixing BITQ and a representative ^10^B-labeled boronoagent, BPA, immediately produced significant fluorescence in a highly quantitative and selective manner. BITQ enabled the visualization of BPA distribution within a living cell and quantified the concentration of BPA in mouse blood to a degree comparable with that of current methods. This study highlights the highly effective properties of BITQ as a versatile fluorescence sensor for analyzing boronic acid agents.

**Abstract:**

Boron neutron capture therapy (BNCT) is an attractive approach to treating cancers. Currently, only one ^10^B-labeled boronoagent (Borofalan, BPA) has been approved for clinical BNCT in Japan, and methods for predicting and measuring BNCT efficacy must be established to support the development of next-generation ^10^B-boronoagents. Fluorescence sensors targeting boronic acids can achieve this because the amount and localization of ^10^B in tumor tissues directly determine BNCT efficacy; however, current sensors are nonoptimal given their slow reaction rate and weak fluorescence (quantum yield < 0.1). Herein, we designed and synthesized a novel small molecular-weight fluorescence sensor, BITQ, targeting boronic acids. In vitro qualitative and quantitative properties of BITQ were assessed using a fluorophotometer and a fluorescence microscope together with BPA quantification in blood samples. BITQ exhibited significant quantitative and selective fluorescence after reacting with BPA (post-to-pre-fluorescence ratio = 5.6; quantum yield = 0.53); the fluorescence plateaued within 1 min after BPA mixing, enabling the visualization of intracellular BPA distribution. Furthermore, BITQ quantified the BPA concentration in mouse blood with reliability comparable with that of current methods. This study identifies BITQ as a versatile fluorescence sensor for analyzing boronic acid agents. BITQ will contribute to ^10^B-boronoagent development and promote research in BNCT.

## 1. Introduction

Boron neutron capture therapy (BNCT) is a cancer treatment method based on the nuclear capture and fission reactions that occur when boron-10 (^10^B) atoms collide with externally irradiated thermal neutrons. This produces high-linear energy transfer (LET) alpha (α) particles (^4^He) and recoiling lithium-7 (^7^Li) nuclei. These high LET radiations have very short ranges (<10 μm), and their destructive effects are therefore limited to ^10^B-containing cells [1,2]. 4-[^10^B]Borono-L-phenylalanine (BPA) is the most representative and extensively studied boronoagent for BNCT [3,4,5]. BPA is delivered to the intracellular space in a cancer cell-specific manner via the L-type amino acid transporter 1 (LAT1) [6,7]. Following positive outcomes in clinical trials, BPA was approved in Japan in 2020 and is commercially available under the name Borofalan (^10^B). However, the application of BPA is limited to the locally advanced or recurrent unresectable head and neck cancer [8], and next-generation ^10^B-boronoagents are urgently needed to expand the applicability of BNCT to a broader cancer treatment field; consequently, several studies are ongoing to fulfill this purpose [9,10,11,12].

Given the therapeutic mechanism of BNCT, the ^10^B concentration in the tumor directly affects the therapeutic effect and must be monitored in clinical settings to estimate the intratumor ^10^B concentration during continuous BPA infusion [13,14]. Furthermore, the subcellular ^10^B localization is known to affect the therapeutic effect, considering the efficiency of DNA double-strand breakage [15,16]. Therefore, it is critical to develop a method to quantify ^10^B concentrations and visualize ^10^B localization in cancers to support the efficient development of novel ^10^B-boronoagents and predict and measure the therapeutic effects of clinical BNCT. Inductively coupled plasma mass spectrometry (ICP-MS) and inductively coupled plasma optical emission spectrometry (ICP-OES) have been used as the standard methods for determining ^10^B concentration from initial research to clinical measurements. However, these methods are not applicable for localization assessment because they require ashing samples using a high concentration of acid [13]. Recently, the visualization of boron localization in tissues has been made possible by applying mass imaging (secondary ion mass spectrometry, SIMS) [17,18] and autoradiography [19]. However, these methods require high-cost equipment and complex pretreatment and data acquisition processes and thus are not feasible to use for quick or quantitative measurements.

Consequently, small molecular-weight compounds that emit fluorescence after reacting with boronic acid have been recognized as fluorescence sensors that can be used to evaluate ^10^B-boronoagents. DAHMI is a well-known fluorescence sensor [10,20,21]. The fluorescence intensity emitted after reacting with boronic acid is sufficient to visualize intracellular boronic acid distribution, and DAHMI has been used to evaluate various BNCT agents to date. However, fluorescence is only slowly produced after the reaction with boronic acid, preventing DAHMI from being used for rapid quantitative measurements. Recently, we reported a novel fluorescence sensor, PPN-1, that reacts quickly with boronic acid [22] but exhibits a low fluorescence quantum yield that could not be applied to fluorescence microscopic observation.

**Figure 1:**
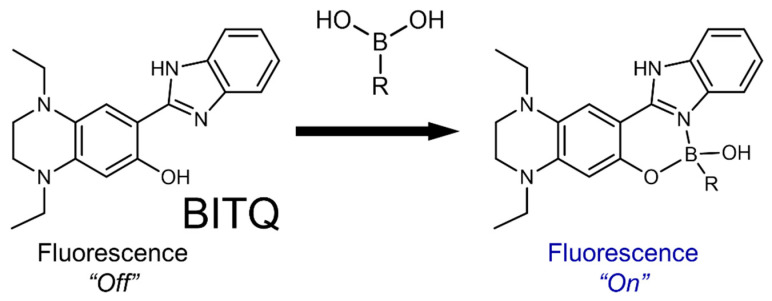
Schematic of boronic acid detection by formation of a fluorescent complex with BITQ.

Herein, we aimed to develop a novel sensor compound that emits strong fluorescence with a high quantum yield immediately after reacting with boronic acid. Specifically, we selected 2-(2-hydroxyphenyl)-1*H*-benzimidazole (HPBI), which rapidly reacts with boronic acid and emits strong fluorescence regardless of forming a complex with boronic acid [23,24] as the skeleton of a fluorescence sensor. To achieve an off/on fluorescence property, we introduced an electron-donating moiety to the skeleton to suppress the fluorescence before complexation with boronic acid [25]. We then adopted the 1,4-diethyl-1,2,3,4-tetrahydropyrazine structure among electron-donating groups to design BITQ (Figure 1) to not only suppress the fluorescence before the complexation but also improve fluorescence properties after the complexation [26,27,28] in comparison with those of the diethylamino group of DAHMI [29].

Therefore, we synthesized BITQ and evaluated its fundamental properties as a boronic acid fluorescence sensor in an aqueous solution compared with those of HPBI and DAHMI. We then applied BITQ to visualize the subcellular boronic acid distribution and to quantify boronic acid in the blood to estimate its potential for assessing ^10^B-boronoagents.

## 2. Materials and Methods

### 2.1. Preparation of BITQ

All reagents were obtained from Tokyo Chemical Industry (Tokyo, Japan), Nacalai Tesque (Kyoto, Japan), or Wako Pure Chemical Industry (Osaka, Japan) and used without further purification. BPA was supplied by Stella Pharma Corp. (Osaka, Japan). Mass spectra (MS) and high-resolution mass spectra (HRMS) were collected with a JMS-700(2) mass spectrometer (JEOL Ltd., Tokyo, Japan). ^1^H and ^13^C-NMR spectra were obtained with a DD2 NMR Spectrometer (Agilent, CA, USA, 600 MHz). An intermediate of BITQ, 1,4-diethyl-7-hydroxy-1,2,3,4-tetrahydro-quinoxaline-6-carbaldehyde, was synthesized as previously described [29]. 1,2-Phenylenediamine (0.03 g, 0.26 mmol) was added to DMF (2 mL) solution containing the intermediate (0.06 g, 0.26 mmol) and stirred for 1 min at room temperature (RT). Na_2_S_2_O_5_ (50.2 mg, 0.26 mmol) in water (0.5 mL) was added to the solution and stirred for 4 h at 90 °C. After completion of the reaction, the reaction mixture was diluted with ethyl acetate (150 mL) and washed with water. The organic layer was dried over anhydrous Na_2_SO_4_ and concentrated under reduced pressure. The residue was purified by silica gel chromatography (hexane/EtOAc) to afford BITQ (0.05 g, 56%) as an orange solid. ^1^H NMR (600 MHz, DMSO-d6): δ1.10 (t, *J* = 7.2 Hz, 3H), 1.14 (t, *J* = 6.6 Hz, 3H), 3.15 (m, 2H), 3.34 (m, 4H), 3.40 (m, 2H), 6.11 (s, 1H), 7.06 (s, 1H), 7.15–7.19 (m, 2H), 7.48–7.50 (m, 1H), 7.55–7.56 (m, 1H), 12.5 (brs, 1H), 12.6 (brs, 1H); ^13^C NMR (150 MHz, DMSO-d6): δ9.7, 10.0, 44.4, 44.7, 44.7, 46.4, 97.4, 99.1, 107.2, 110.4, 116.6, 121.5, 121.7, 127.8, 133.0, 139.3, 141.4, 152.7, 153.5, FAB-MS: *m/z*: 322. Measured: 323[+H]^+^. FAB-HRMS: Calculated for C_19_H_23_N_4_O: 323.1872. Measured: 323.1868.

### 2.2. Fluorescence Properties

BITQ was mixed with BPA to final concentrations of 1.0 and 100 μM, respectively, in 0.5% DMSO/H_2_O. Excitation and emission spectra were measured 15 min later using a spectrometer (FP-8600, JASCO Corporation, Tokyo, Japan, photomultiplier voltage: 700 V) to evaluate the maximum excitation ($\lambda_{\max}^{ex}$) and emission ($\lambda_{\max}^{em}$) wavelengths. HPBI was analyzed similarly. The fluorescence intensity (λex/λem: 390/480 nm; photomultiplier voltage: 700 V) was intermittently measured for 30 min after the addition of BPA (100 μM) to BITQ (1.0 μM) in 0.5% DMSO/H_2_O.

To evaluate the relationship between the fluorescence intensity and BPA concentration, BITQ was mixed to a final concentration of 1.0 μM with various concentrations of BPA (0–50 μM at final concentration) in 0.5% DMSO/H_2_O. Fluorescence intensities were measured using a spectrometer 15 min after mixing (λex/λem: 390/480 nm). A linear regression analysis was performed to fit the data (0–10 μM, n = 5) and calculate the quantification and detection limits from the following equations:

quantification limit = 10 σ/slope and detection limit = 3 σ/slope,

where σ is the standard deviation of the fluorescence intensities of samples at 0 µM, and the slope is derived from the regression line. DAHMI was mixed to a final concentration of 1.0 mM with various concentrations of BPA (0–40 μM at final concentration) in 50% DMSO/H_2_O. Fluorescence intensities were measured using a spectrometer 120 min after mixing (λex/λem: 411/431 nm, photomultiplier voltage: 1130 V) and analyzed similarly.

The relative quantum yields of BITQ, HPBI, and DAHMI (λex = 330, 310, and 330 nm, respectively) were measured in ethanol before and after mixing with phenylboronic acid (50 eq.) using anthracene (φ_R_ = 0.27) as a reference following the equation:

φ_S_ = φ_R_ × (Abs_R_/Abs_S_) × (Area_S_/Area_R_),

where the subscripts R and S refer to the reference and sample, respectively, and Abs and Area refer to the absorbance at the excitation wavelength and the area under the fluorescence spectrum, respectively.

### 2.3. Reactivity for Boron-Containing Compounds

BITQ (final concentration of 1.0 μM) was mixed with boric acid, ethylboronic acid, 2-(hydroxymethyl)phenyl boronic acid monoester, or bis(pinacol)diboron (final concentration of 100 μM) in 0.5% DMSO/H_2_O. Fluorescence intensities were measured using a spectrometer 5 min after mixing (photomultiplier voltage: 700 V).

### 2.4. Selectivity Assay

BITQ (final concentration of 1.0 μM) was mixed with BPA or a metal cation (NaCl, KCl, MgCl_2_·6H_2_O, CaCl_2_, FeCl_2_·4H_2_O, FeCl_3_·6H_2_O, CoCl_2_·6H_2_O, ZnCl_2_, CdCl_2_·2.5H_2_O, NiCl_2_·6H_2_O, CuCl_2_, MnCl_2_·4H_2_O, or AlCl_3_·6H_2_O) (final concentration of 100 μM) in 0.5% DMSO/Tris HCl buffer (100 mM, pH 7.4). Fluorescence intensities of the samples were measured 15 min after mixing using a plate reader (EnSpire Multilabel Reader 2300, PerkinElmer Japan, Kanagawa, Japan, λex/λem: 390/480 nm) in quadruplicate (n = 3). The fluorescence intensities were expressed in relative value to that of the BITQ solution in the absence of metal cations and BPA.

BITQ, BPA, and each metal cation above (final concentration of 1.0, 100, and 100 μM, respectively) were mixed in 0.5% DMSO/Tris HCl buffer (100 mM, pH 7.4) in quadruplicate (n = 3), followed by the measurement of fluorescence intensities at 15 min as above. Fluorescence intensities were expressed relative to that of the BITQ-BPA solution without adding metal cations.

### 2.5. Fluorescence Microscopy

T3M-4 human pancreatic adenocarcinoma cells (RCB1021) were provided by the RIKEN BioResource Research Center (Ibaraki, Japan) and were cultured in DMEM/Ham’s F-12 medium containing 10% fetal bovine serum at 37 °C in a humidified atmosphere of 5% CO_2_. The uptake of BPA into T3M-4 cells was performed with several modifications to the previously reported method [29]. Briefly, T3M-4 cells were cultured in a glass bottom dish 35 mm (Matsunami Glass Ind., Osaka, Japan) 2 days before the experiment. After washing three times with Na^+^-free Hank’s balanced salt solution (HBSS: 125 mM choline chloride, 25 mM HEPES, 4.8 mM KCl, 5.6 mM D-glucose, 1.3 mM CaCl_2_, 1.2 mM MgSO_4_, and 1.2 mM KH_2_PO_4_), 1.5 mL of BPA (1.0 mM in HBSS) was added and incubated at 37 °C for 30 min. For the BPA-absent group, only HBSS was added and incubated. After washing three times with HBSS, 1.5 mL of BITQ (10 μM in 0.5% DMSO/HBSS) was added and incubated for 5 min at RT. For nuclei staining, cells were incubated with NucRed Live647 (Thermo Fisher Scientific, Tokyo, Japan, 1:15 dilution in HBSS) at 37 °C for 30 min. Fluorescence images were acquired with a BZ-X810 (Keyence, Osaka, Japan) instrument using a DAPI-V filter cube (Keyence OP-88359, Ex: 395/25 nm, Em: 460/50 nm) and a Cy5 filter (Keyence OP-87766, Ex: 620/60 nm, Em: 700/75 nm) for BITQ and NucRed Liver647, respectively.

### 2.6. Quantification of BPA Concentration in Mice Plasma

Male ddY mice (6–8 weeks old, Japan SLC, Shizuoka, Japan) were housed under a 12 h light/12 h dark cycle and given free access to food and water. Animal experiments were conducted according to the institutional guidelines for animal experiments. The study protocol was approved by the institutional Experimental Animal Committee (Permission Number: 21-76 and 22-76). To solubilize BPA, BPA and 2.2 equivalents of D-fructose were pre-mixed to make a BPA-fructose solution. BPA-fructose solution (20 µL; final concentration of 0–900 µM) was added to blood (480 µL) collected from mice and mixed by inversion. After 5 min, plasma was collected by centrifugation. Methanol (400 µL) was added to the plasma (200 µL), vortexed for 30 s, and centrifuged to remove protein. A 100-µL volume of the supernatant was added to 2.9 mL 0.5% DMSO/H_2_O solution containing BITQ (final 5.0 µM), followed by the fluorescence measurement using a spectrometer 15 min after mixing (λex/λem: 390/480 nm, photomultiplier tube voltage: 700 V). A linear regression analysis was performed to fit the data (n = 3).

To evaluate the quantitative range of the fluorescence analysis with BITQ compared with that of ICP-MS, mouse blood (480 µL) was mixed with an unknown concentration of BPA-fructose solution (20 µL) prepared blindly. The samples were divided into two parts and measured by the lead researcher (S.T.) using fluorescence analysis with BITQ and ICP-MS. For ICP-MS measurement, the sample was ashed with concentrated nitric acid, and the boron concentration was determined using 8800 triple quadrupole ICP-MS (Agilent, Santa Clara, CA, USA).

### 2.7. Statistics

Data are presented as mean ± standard deviations. Statistical analyses were performed using Dunn’s multiple comparison tests with GraphPad Prism 8. Differences at the 95% confidence level (*p* < 0.05) were considered significant.

## 3. Results

### 3.1. Fluorescence Properties of BITQ

**Figure 2:**
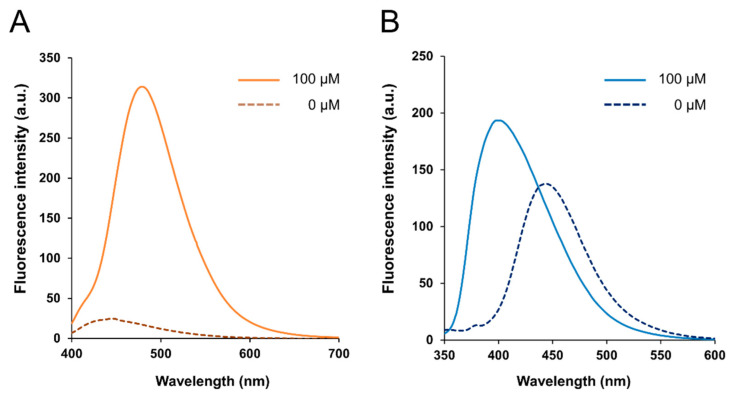
Fluorescence spectra of (**A**) BITQ (1.0 µM) and (**B**) HPBI (1.0 µM) 15 min after the addition of BPA (0 or 100 µM) in 0.5% DMSO/H_2_O. (λex: HPBI; 337 nm, BITQ; 390 nm).

BITQ was synthesized with a yield of 56.3%. The fluorescence properties of BITQ, HPBI, and DAHMI are summarized in Table 1. After the addition of BPA, the maximum excitation ($\lambda_{\max}^{ex}$) and emission wavelengths ($\lambda_{\max}^{em}$) of BITQ fluorescence were 390 and 480 nm, respectively (Figure 2A), showing the longer emission wavelength and broader Stokes shift compared with those of HPBI ($\lambda_{\max}^{ex}$: 337 nm; $\lambda_{\max}^{em}$: 399 nm; Figure 2B). The quantum yield of HPBI before the addition of boronic acid was 0.71, but this was significantly suppressed to 0.09 for BITQ. Consequently, the post-to-pre-fluorescence ratio was 5.6 and 1.3 for BITQ and HPBI, respectively, indicating the superior off/on property of BITQ. The quantum yield of BITQ after adding boronic acid was 0.53, which was ten-fold larger than that of DAHMI (0.053). Aside from BPA, the fluorescence increased markedly after mixing BITQ with 2-(hydroxymethyl)phenyl boronic acid monoester, but the increase in fluorescence was slight for boric acid, ethylboronic acid, and bis(pinacol)diboron, which do not contain a benzene ring (Figure S1).

**Figure 3:**
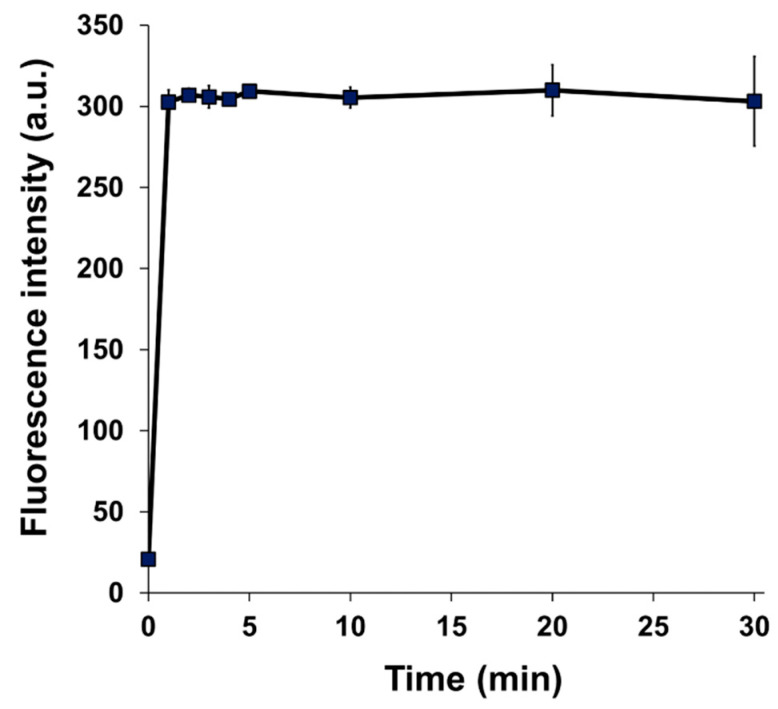
Temporal change in fluorescence intensity after mixing of BITQ (1.0 µM) and BPA (100 µM) in 0.5% DMSO/H_2_O (λex = 390 nm, λem = 480 nm).

**Figure 4:**
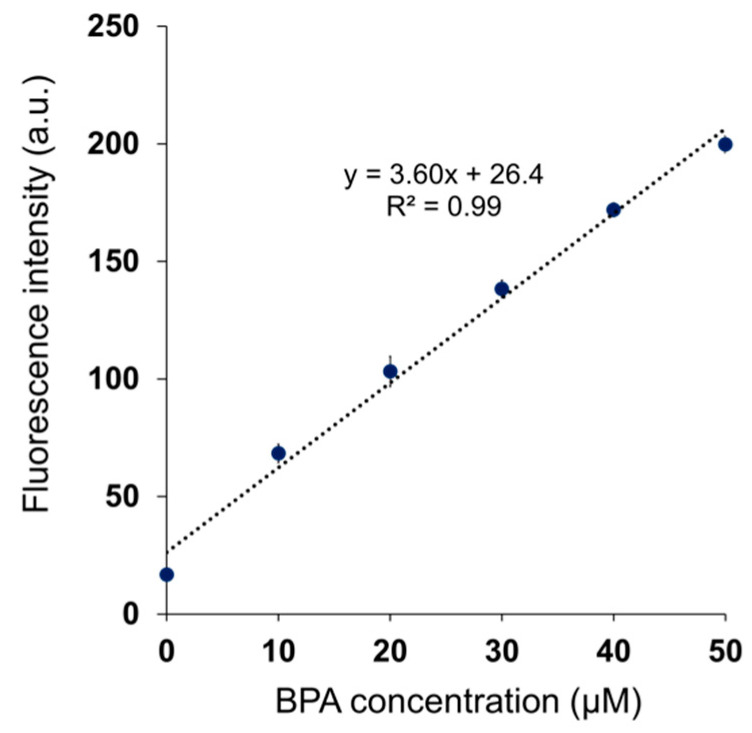
Linear regression analysis between the fluorescence intensities of BITQ (1.0 µM) and BPA concentrations treated (0–50 µM) in 0.5% DMSO/H_2_O. (λex = 390 nm, λem = 480 nm).

The fluorescence intensity of BITQ increased within 1 min after adding BPA and remained constant for 30 min (Figure 3). For DAHMI, the fluorescence intensity did not plateau until 120 min after BPA addition [22], suggesting a faster reactivity of BITQ with BPA compared with that of DAHMI. Linear regression analysis revealed high linearity (R^2^ = 0.99, Figure 4) between the fluorescence intensities of BITQ and the concentrations of BPA reacted. The detection and quantification limits were 0.24 and 0.82 µM, respectively (R^2^ = 0.99, Figure S2). As for DAHMI, the detection and quantification limits were 0.72 and 2.40 µM, respectively (R^2^ = 0.99, Figure S3).

### 3.2. Selectivity Assay

**Figure 5:**
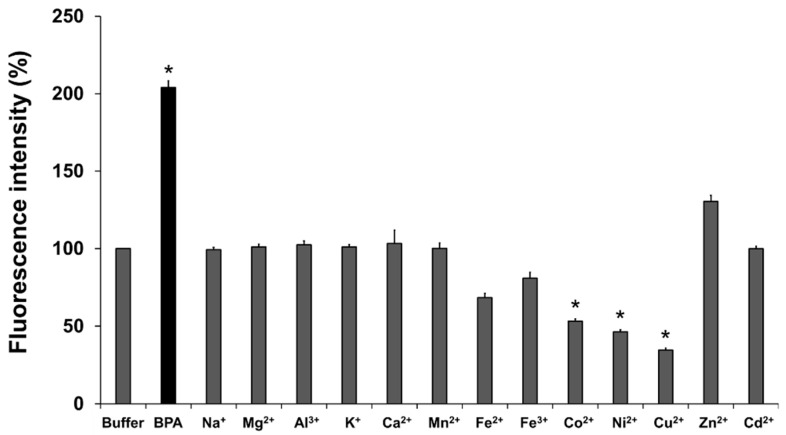
BITQ fluorescence intensity (1.0 μM) 15 min after adding BPA or metal cation (100 μM, pH 7.4) with buffer-only sample as a standard (100%). * *p* < 0.05 vs. buffer (0.5% DMSO/Tris HCl buffer only) by Dunn’s multiple comparison test.

The changes in the fluorescence of BITQ (1.0 μM) after adding various metal cations (100 μM) are summarized in Figure 5. The BITQ fluorescence significantly increased after adding BPA (>200%) but did not increase after adding any of the metal cations tested except for Zn^2+^ (130%, not significant).

Next, the effects of the coexistence of high concentrations of metal cations (100 μM) in the fluorescence intensity of the solution containing BITQ (1.0 μM) and BPA (100 μM) were evaluated (Figure S4). Lower fluorescence was evident when Mn^2+^, Fe^2+^, Fe^3+^, Co^2+^, Ni^2+^, and Cu^2+^ coexisted in the BITQ/BPA solution compared with that of the control.

### 3.3. Fluorescence Microscopy Study

**Figure 6:**
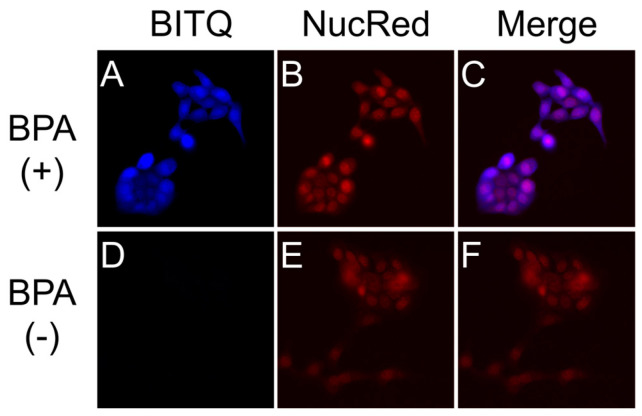
Representative fluorescence images of T3M-4 cells: (**A**–**C**) BPA-present group, (**D**–**F**) BPA-absent group. (**A**,**D**) Fluorescence images after 5 min incubation with BITQ (10 μM) (DAPI-V filter: Ex: 395/25 nm, Em: 460/50 nm); (**B**,**E**) nucleus staining using NucRed Live647 (Cy5 filter: Ex: 620/60 nm, Em: 700/75 nm); and (**C**,**F**) merged images of fluorescence from BITQ and NucRed Live647.

Fluorescence images of T3M-4 cells pretreated with or without BPA after adding BITQ are shown in Figure 6A,D (pseudo-color; blue). Faint fluorescence was observed in the BPA-absent group (Figure 6D), while fluorescence was observed throughout the cells in the BPA-treated group (Figure 6A). The merged image (Figure 6C) indicated that the BITQ fluorescence was strongly present around the cell nuclei visualized with NucRed Live647 (Figure 6B, pseudo-color; red).

### 3.4. Quantification of BPA Concentration in Mouse Blood

**Figure 7:**
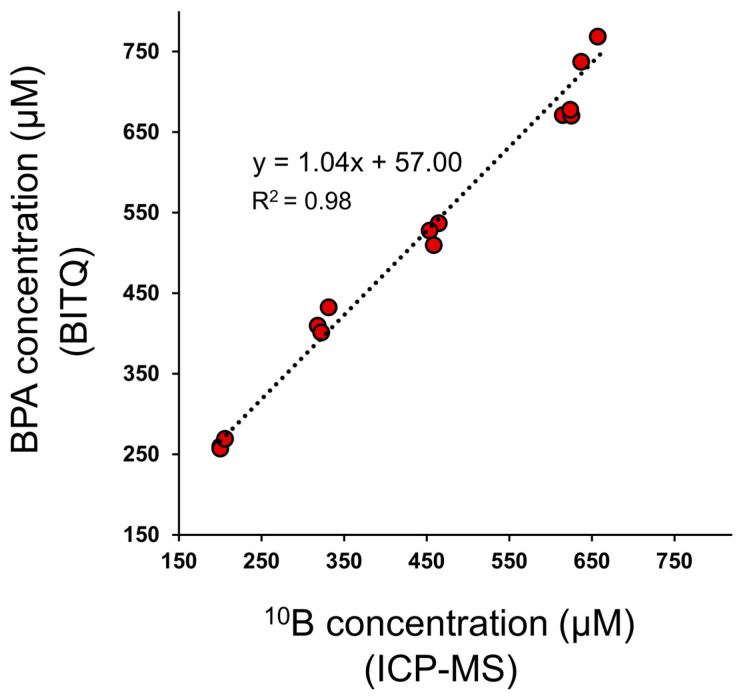
Linear regression analysis between the ^10^B concentration determined by ICP-MS and BPA concentration determined by fluorescence analysis using BITQ in the same mouse blood.

Linear regression analysis revealed that the BITQ fluorescence was obtained with high linearity over a wide range of the BPA concentrations in mice blood (R^2^ = 0.99, Figure S5). Furthermore, in an experiment using blood samples containing BPA blindly prepared for the authors, the boron concentration measured from the BITQ fluorescence was linearly correlated with that evaluated by the ICP-MS analysis with a slope of 1 (R^2^ = 0.98, Figure 7).

## 4. Discussion

Herein, we synthesized a novel off/on-type fluorescence sensor (BITQ) that targeted boronic acids and was designed by introducing the 1,4-diethyl-1,2,3,4-tetrahydropyrazine structure as an electron-donating group [26,27,28] to the HPBI skeleton to improve the fluorescence properties. We then evaluated the potential of BITQ as a sensor compound for qualitative and quantitative analyses in the BNCT-related fields. As expected, we found that BITQ had promising properties, including a high quantum yield, high off/on switching ability, rapid reactivity, longer emission wavelength, and extended Stokes shift, in comparison with these aspects of DAHMI used as a control [20,21,22]. These results indicate that BITQ can potentially replace DAHMI to promote the development of ^10^B-boronoagents and evaluate BPA in BNCT research.

The high quantum yield and off/on ability of BITQ produced an improved quantification limit (0.82 μM) that was superior to those of DAHMI (2.40 μM), PPN-1 (6.01 μM) [22], and BS-631 (65.3 μM) [29], which we previously reported as a sensor for BNCT, suggesting a high sensitivity comparable with that of ICP-OES [30] and that would be sufficient for measuring the boron concentration (>2 mM) required for BNCT [2]. The BITQ fluorescence rapidly increased to maintain a constant value for 30 min after mixing with BPA, indicating the high stability in an aqueous solution as well as the high reactivity mentioned above. DAHMI contains (E)-2-[(methylimino)methyl]phenol as the core structure to react with boronic acids, although this produces instability in an aqueous solution in addition to the slow reactivity [31]; therefore, BITQ would be more efficacious than DAHMI for quantitative assessment during in vitro pharmacokinetic studies such as a time-dependent uptake experiment for evaluating the effect of ^10^B-boronoagents. The subcellular ^10^B localization is known to affect the efficiency of energy transfer to the nucleus [15,16,32]. In addition, the intracellular trafficking and the efficiency of efflux of agents differ depending on the localization position (organelle) after intracellular uptake of the agent [10]. Therefore, it is significant to visualize ^10^B localization in cancers. In this study, BPA distribution in high LAT1-expressing T3M-4 cells [7,9] was visualized using fluorescence microscopy with BITQ at a lower concentration and in a shorter time (10 μM, 5 min) compared with the conditions reported for DAHMI (1 mM, 20 min) [20], which was attributable to the higher quantum yield, faster reactivity, and improved adaptability to filter sets due to Stokes shift expansion. The intracellular BPA distribution seen in this study was visually consistent with previously reported localization evaluated by SIMS [17,18]. BITQ could also be applied to microscopic fluorescence observation using living cells, unlike DAHMI, which requires a fixation process for the observation. Thus, BITQ is expected to be applied to observing changes in boron uptake over time in living cells and could be a powerful tool for drug development for BNCT.

BITQ fluorescence did not increase following mixing with major cations known to be present at millimolar levels in cells [33]. However, Zn^2+^ did react with BITQ to produce a small, but not significant, increase in fluorescence. The fluorescence intensity of BPA/BITQ was attenuated by up to approximately 20% for metal ions (Fe^2+^, Fe^3+^, Co^2+^, Ni^2+^, and Cu^2+^) when 100 equivalents of metal ions were added to the sensor. A similar tendency was observed with the fluorescence of DAHMI and PPN-1 in the presence of trace metals [22]. To achieve BNCT treatment, intratumor boron concentration was required to be 2 mM or higher. In contrast, the amount of intracellular metal ions present is reportedly at the sub-micromolar or few micromolar levels [34]. Therefore, the influence of coexisting trace metal cations is negligible in the detection ability of BITQ.

Currently, ^18^F-labeled BPA ([^18^F]FBPA, 4-Borono-2-[^18^F]fluoro-L-phenylalanine) is utilized as a positron emission tomography (PET) probe for imaging and the evaluation of the pharmacokinetics of BPA in vivo [35]. In clinical BNCT using BPA, [^18^F]FBPA-PET is performed before BNCT treatment. The tumor-to-normal and tumor-to-blood ratios of radioactivity accumulation are calculated from the PET images to determine whether BNCT is applicable. Since the amount of boron accumulated in the tumor is estimated from the tumor-to-blood ratio data obtained from PET and the actual blood boron concentration during treatment, it is necessary to quantify the ^10^B concentration in the venous blood via ICP-MS/OES method during 2–3 h BPA infusion several times [14,36]. Therefore, ICP-MS/OES is indispensable equipment for hospitals currently performing BNCT. As BNCT continues to expand, the number of BNCT hospitals is expected to increase. In such case, fluorescence measurement using a plate reader is considered safer and less difficult to perform than the ICP-MS/OES method, which requires hazardous treatment with high concentrations of acid. We thus tried to quantify BPA concentrations in mouse blood using BITQ because fluorescence analysis is generally used in clinics, reasonable to install, and easy to perform by clinicians. The results clearly showed the effectiveness of BITQ in quantifying BPA in the blood with high precision comparable with that of the ICP-MS method within 30 min after blood collection. Although further efforts are required to shorten the measurement protocol to apply to actual clinical practice, this result strongly suggests that the ex vivo fluorescence analysis with BITQ can be an alternative BPA quantification method in patient blood in the future.

We mainly used BPA as a boronic acid-containing compound to validate the effectiveness of BITQ, as BPA is currently the only ^10^B-boronoagent available for clinical BNCT. Boronic acid-containing structures are recognized as having potential for the development of effective therapeutic agents [37,38,39], including ^10^B-boronoagents for BNCT [9,10,11,12]. This study has provided preliminary evidence that BITQ exhibited a marked increase in fluorescence after reaction with boronic acids containing a benzene ring, such as BPA, phenylboronic acid, and 2-(hydroxymethyl)phenyl boronic acid monoester, while the fluorescence increase was attenuated with structures that do not contain benzene rings, such as boric acid and ethylboronic acid. Thus, BITQ will need further careful assessment for evaluating its reactivity to a boronoagent newly developed in the future. Furthermore, although it is reasonably expected that BITQ will be applicable to ex vivo samples, which SIMS and autoradiography can assess [17,18,19], this should also be validated. For example, BITQ would be a powerful tool to visualize ^10^B distribution in ex vivo tumor sections in preclinical experiments for predicting the therapeutic effect of drug candidates for BNCT.

## 5. Conclusions

In this study, we developed a novel fluorescence sensor targeting boronic acids, BITQ, showing high quantum yield, excellent off/on ability, rapid reactivity, and high quantitative scope for BPA. BITQ could visualize intracellular BPA in a short time and quantify BPA in mice blood with a precision comparable with that of ICP-MS. This study highlights the excellent properties of BITQ as a versatile fluorescence sensor for analyzing boronic acid agents, and BITQ will therefore contribute to developing ^10^B-boronoagents and promote biological research in BNCT.

## Figures and Tables

**Table 1:** Fluorescence properties of tested compounds.

	$\lambda_{max}^{ex}$	$\lambda_{max}^{em}$	Stokes Shift	φboron	φfree	φboron /φfree
BITQ	390 nm	480 nm	90 nm	0.53	0.094	5.6
DAHMI	411 nm	431 nm	20 nm	0.053	0.0034	15.7
HPBI	337 nm	399 nm	62 nm	0.89	0.71	1.3

## Data Availability

The data supporting the results and findings of this study are available within the paper and the Supplementary Information files. Additional raw data are available from the corresponding author upon request.

## Supplementary Materials

The following supporting information can be downloaded at: https://www.mdpi.com/article/10.3390/cancers15061862/s1, Figure S1: The structure of tested boron-containing compounds and the emission spectra of BITQ after the addition of these compounds; Figure S2: Linear regression analysis between the fluorescence intensities of BITQ (1.0 µM) and BPA (0–10 µM); Figure S3: Linear regression analysis between the fluorescence intensities of DAHMI (1.0 mM) and BPA (0–40 µM); Figure S4: BITQ fluorescence intensity (1.0 μM) 15 min after the addition of BPA (100 μM) when coexisted with metal cations (100 μM, pH 7.4); Figure S5: Linear regression analysis between the fluorescence intensities of BITQ (5.0 µM) and BPA concentrations contained (0–900 µM) in mouse blood.
