# Supplementary material for: Development of a 2-(2-Hydroxyphenyl)-1H-benzimidazole-Based Fluorescence Sensor Targeting Boronic Acids for Versatile Application in Boron Neutron Capture Therapy
Source: Cancers (Basel). 2023 Mar 20;15(6):1862. doi: 10.3390/cancers15061862 (PMC10046934; doi:10.3390/cancers15061862)
Supplement: Supplementary file 1 [file cancers-15-01862-s001.zip › cancers-2212555-supplementary.pdf]

cancers

MDPI

Supplemental information

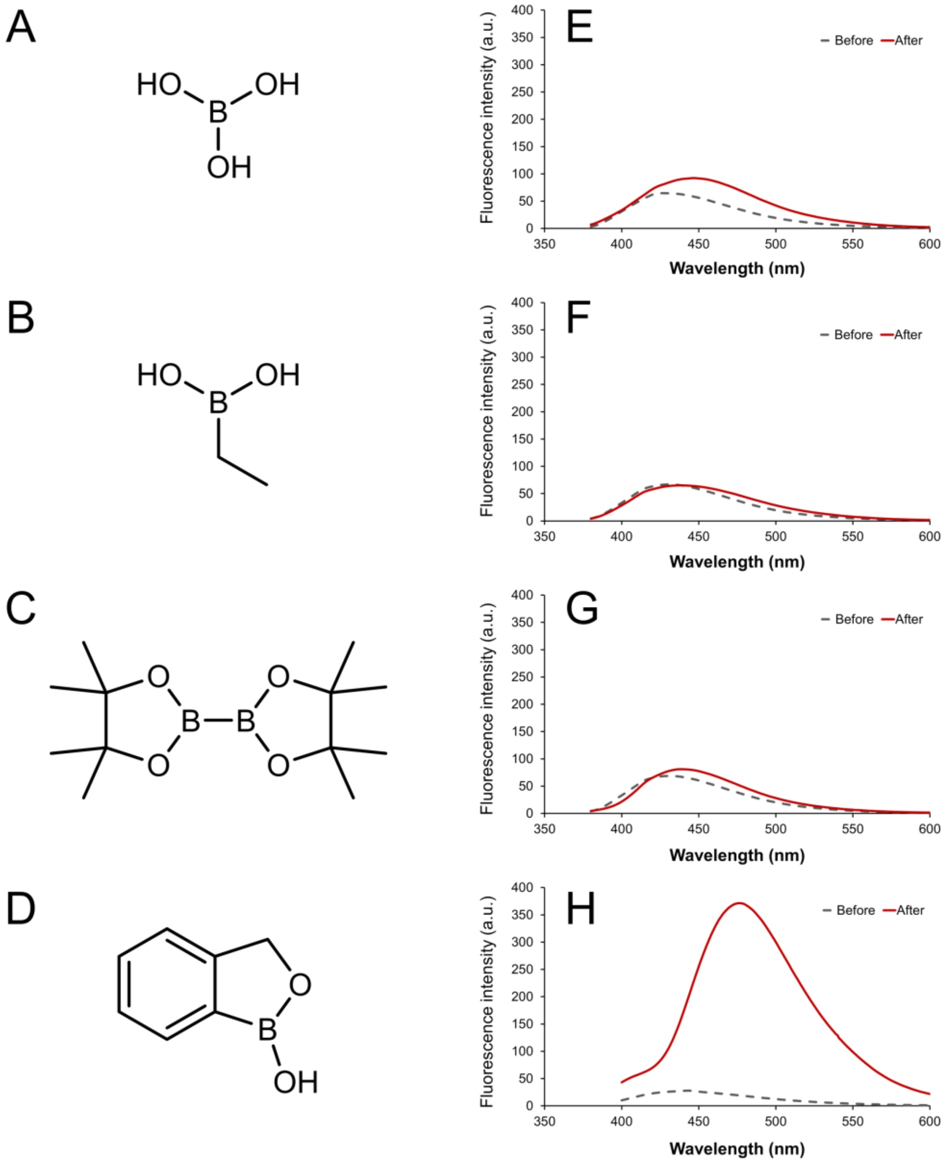

Figure S1. The structure of tested boron-containing compounds (A-D) and the emission spectra of BITQ 5 min after addition of these compounds (E-H); Boric acid (A, E; λex = 371 nm), ethylboronic acid (B, F; λex = 365 nm), bis(pinacol)diboron (C, G; λex = 367 nm), and 2-(hydroxymethyl)phenyl boronic acid monoester (D, H; λex = 388 nm)

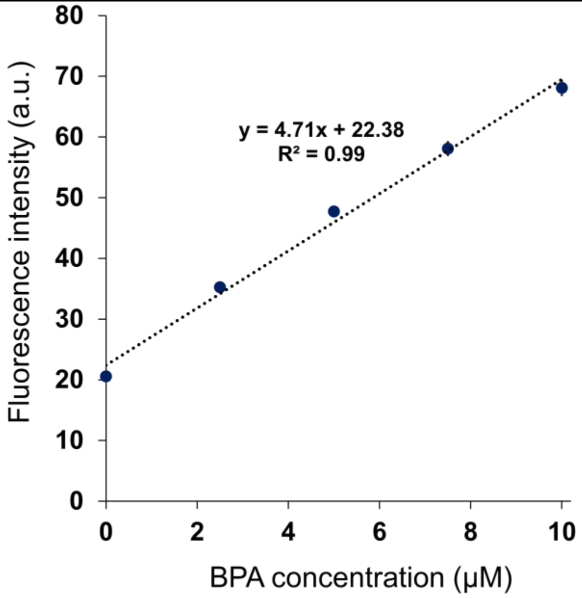

Figure S2. Linear regression analysis between the fluorescence intensities of BITQ (1.0 μM) and BPA concentrations treated (0–10 μM) in 0.5% DMSO/H_2O. (λex = 390 nm, λem = 480 nm).

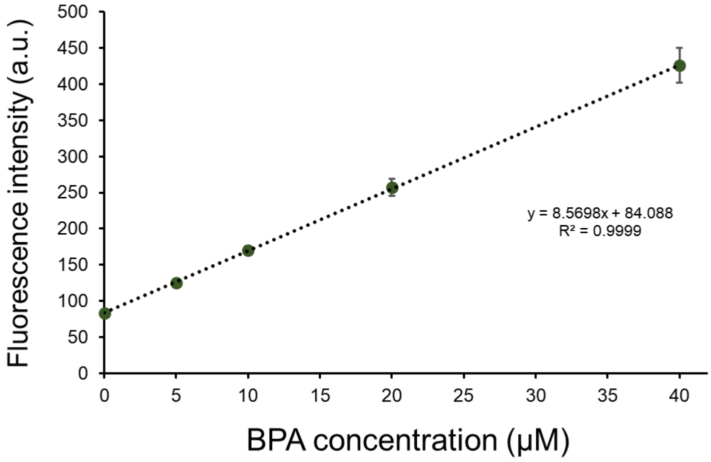

Figure S3. Linear regression analysis between the fluorescence intensities of DAHMI (1.0 mM) and BPA concentrations treated (0–40 μM) in 50% DMSO/H_2O. (λex = 411 nm, λem = 431 nm).

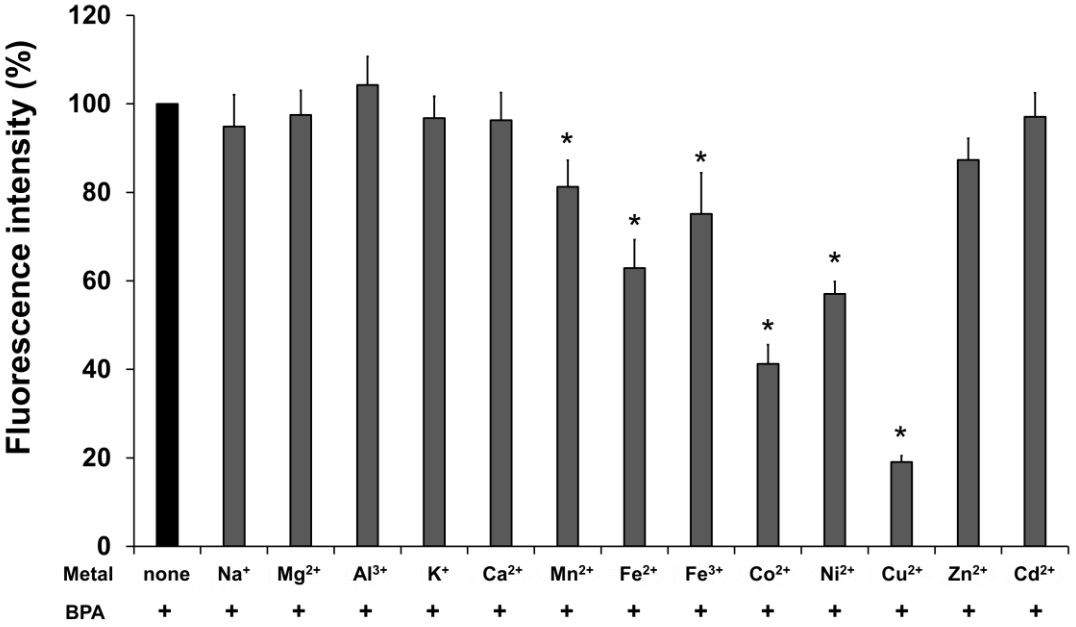

Figure S4. BITQ fluorescence intensity (1.0 μM) 15 min after the addition of BPA (100 μM) when coexisted with metal cations (100 μM, pH 7.4) with BPA-only sample as a standard (100%). *$p < 0.05$ vs. none (BPA only) by Dunn's multiple comparison test.

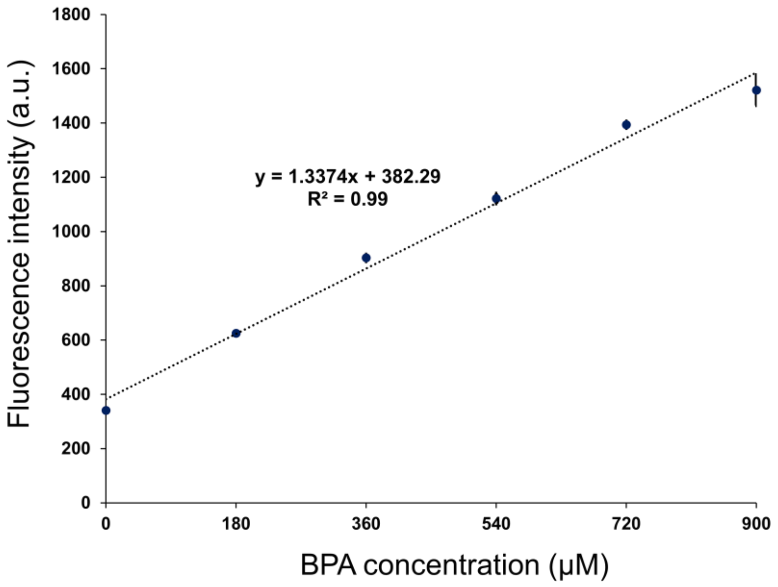

Figure. S5. Linear regression analysis between the fluorescence intensities of BITQ (5.0 μM) and BPA concentrations contained (0–900 μM) in mouse blood (λex = 390 nm, λem = 480 nm).
